# Characterizing the academic literature on surgical management of benign prostatic hyperplasia: A bibliometric analysis

**DOI:** 10.1007/s00345-026-06257-9

**Published:** 2026-02-11

**Authors:** Atınç Tozsin, Erkan Arslan, Osman Ermiş, Burak Akgül, Selçuk Güven, Thomas RW Herrmann

**Affiliations:** 1 Department of Urology, Şırnak Silopi State Hospital, Şırnak, Turkey; 2 Meram School of Medicine, Department of Urology, Necmettin Erbakan University, Konya, Turkey; 3 Department of Urology, İstanbul Avcılar Murat Kölük State Hospital, İstanbul, Turkey; 4 Department of Urology, Indiana University School of Medicine, Indianapolis, IN USA; 5 Department of Urology, Spital Thurgau AG, Kantonsspital Frauenfeld, Pfaffenholzstrasse 4, Frauenfeld, CH Switzerland; 6 Division of Urology Department of Surgical Sciences, Stellenbosch University, Stellenbosch, Western Cape South Africa; 7 Hannover Medical School, Hannover, Germany

**Keywords:** Benign prostatic hyperplasia, BPH management, Bibliometric analysis, Lower urinary-tract symptoms

## Abstract

**Purpose:**

Historically managed with open surgery, benign prostatic hyperplasia (BPH) treatment has evolved significantly with the advent of less invasive procedures aimed at improving safety and recovery. We conducted a bibliometric study using the Web of Science Core Collection to analyze global publication trends related to surgical management of BPH. Our aim was to identify leading authors, institutions, countries, influential journals, and evolving research themes to guide future investigations.

**Methods:**

A bibliometric study was performed using the Web of Science Core Collection (WoSCC) as the primary database. Only original articles and reviews published in English were eligible for inclusion. VOSviewer (v1.6.20) was used to generate co-authorship, country collaboration, and keyword co-occurrence maps. CiteSpace (v6.3.R1) was applied for detecting citation bursts and thematic shifts, while the Bibliometrix package in R (v4.2.1) was utilized for descriptive statistics, Bradford’s Law analysis, and network visualizations. Simple linear regression was applied as a descriptive tool to indicate the overall direction of publication growth and was not intended to model non-linear innovation dynamics or provide predictive inference.

**Results:**

Between January 2016 and August 2025, a total of 2,613 publications on surgical management of BPH were identified. Among these, 2,161 were original articles and 452 were reviews, published across 394 different journals. The annual growth rate was calculated at 2.9%, confirming a steady increase in research output during the study period. Each paper had an average of 3.97 authors, with a mean citation rate of 10.7 per document. The dataset contained 24,415 references, while 24.0% of publications involved international collaborations.

**Conclusion:**

This analysis offers a comprehensive view of global research efforts in surgical BPH management, identifying high-yield topics, influential actors, and emerging modalities. This evolving literature base reflects a broader trend toward individualized, minimally invasive care in urology, with BPH management serving as an example.

**Supplementary Information:**

The online version contains supplementary material available at 10.1007/s00345-026-06257-9.

## Introduction

Benign prostatic hyperplasia (BPH) is one of the most prevalent nonmalignant urological conditions globally, affecting aging men and significantly contributing to lower urinary tract symptoms (LUTS). As of 2021, there were over 112 million prevalent cases worldwide, with projections indicating an increase to over 136 million by 2035 [1]. A systematic review estimated the lifetime prevalence of BPH to be approximately 26.2%, highlighting its widespread impact [2]. The disease burden is especially prominent among men aged 65–69 and continues to rise with increasing life expectancy [3].

Historically managed with open surgery, BPH treatment has evolved significantly with the advent of less invasive procedures aimed at improving safety and recovery. Transurethral resection of the prostate (TURP) became the gold standard, but recent decades have introduced effective alternatives like holmium laser enucleation (HoLEP), photoselective vaporization (PVP), UroLift, Rezum, and Aquablation—offering reduced morbidity and strong clinical outcomes in appropriate patients [4, 5]. Technological innovation has played a transformative role in BPH surgery. Modern laser platforms such as holmium and thulium lasers, along with systems like GreenLight, offer effective and safer alternatives to TURP, especially for larger prostates [6]. Office-based, minimally invasive procedures like UroLift and Rezum have gained traction for their short recovery time, reduced anesthesia need, and preservation of sexual function [7, 8]. Robotic waterjet systems such as Aquablation are also emerging as effective options with sustained outcomes [9]. The integration of artificial intelligence in diagnostics and treatment planning is still in early stages but holds potential to personalize care in the near future.

Although bibliometric analyses focusing on specific surgical techniques (particularly endoscopic enucleation of the prostate) have been published [10], a comprehensive bibliometric evaluation encompassing the full spectrum of surgical treatments for benign prostatic hyperplasia remains lacking. It limits our understanding of the field’s evolution, key contributors, and emerging priorities. To address this, we conducted a bibliometric study using the Web of Science Core Collection (WoSCC) to analyze global publication trends related to surgical management of BPH. Our aim was to identify leading authors, institutions, countries, influential journals, and evolving research themes to guide future investigations.

## Materials and methods

**Fig. 1:**
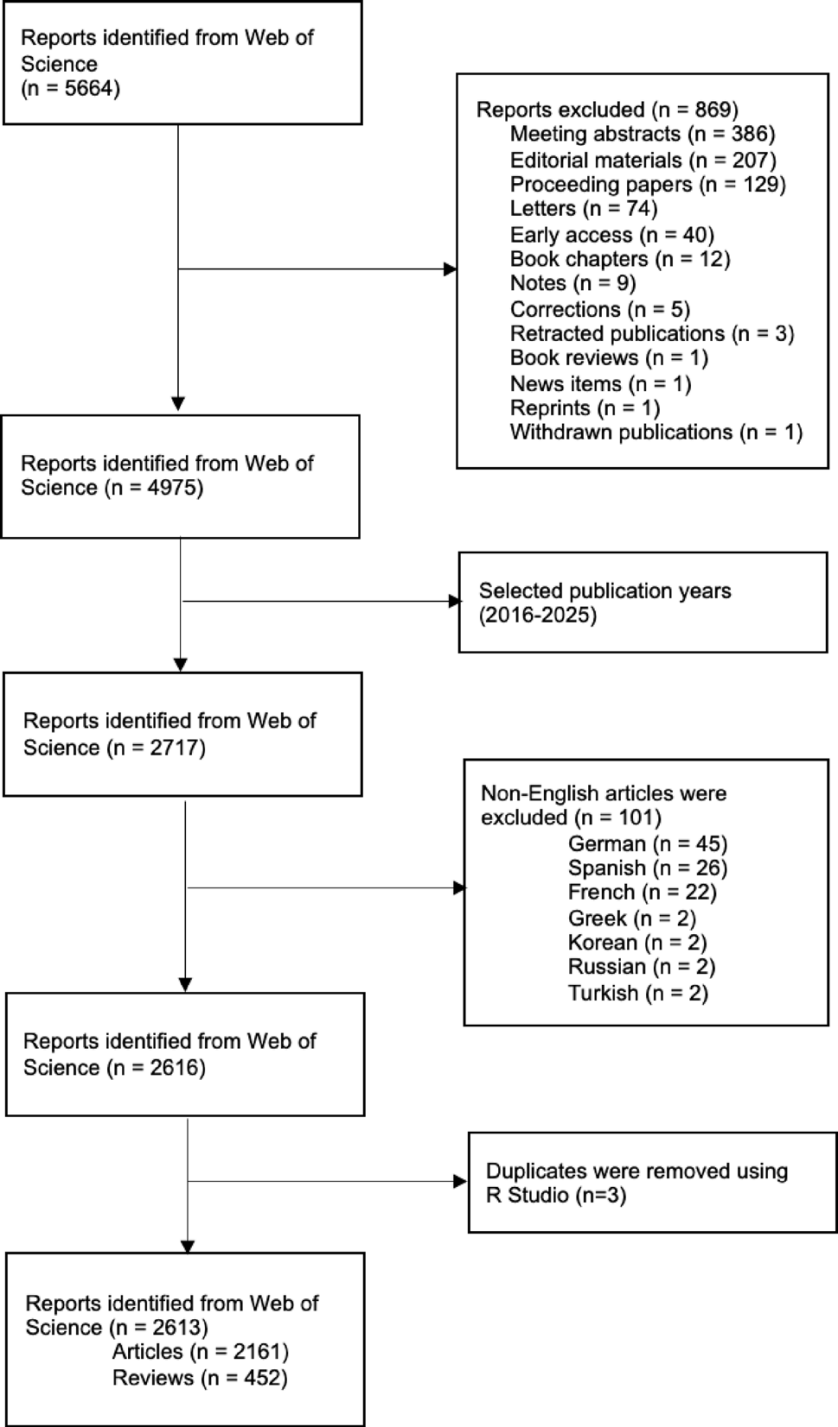
Flowchart of the study

A bibliometric study was performed using the WoSCC as the primary database [11]. The search was conducted on August 18, 2025, and covered the period from January 2016 to August 2025. To capture the surgical management of benign prostatic hyperplasia (BPH), Boolean strategy was applied, including classical procedures such as transurethral resection of the prostate (TURP) and incision of the prostate (TUIP), laser-based interventions such as holmium laser enucleation (HoLEP), thulium laser enucleation (ThuLEP), GreenLight enucleation (GreenLEP), and diode laser enucleation (DiLEP), bipolar resection or enucleation techniques, as well as minimally invasive surgical therapies such as Aquablation, Rezūm, prostatic urethral lift (Urolift), and prostatic artery embolization (PAE). Additionally, laparoscopic and robot-assisted simple prostatectomy and emerging device-based therapies were included. Figure 1 shows the flow diagram of the methodology, and the full search strategy is provided in Supplement 1.

Only original articles and reviews published in English were eligible for inclusion. Editorials, letters, conference abstracts, early access items without full bibliographic information, and non-English publications were excluded. Duplicates (*n* = 3) were removed using R Studio (Version 2025.05.1 + 513), and only studies containing sufficient metadata—such as authorship, country, institutional affiliation, keywords, references, and citation counts—were retained for analysis.

Bibliographic data were exported in plain text format and analyzed using multiple platforms to ensure robustness. VOSviewer (v1.6.20) was used to generate co-authorship, country collaboration, and keyword co-occurrence maps [12]. CiteSpace (v6.3.R1) was applied for detecting citation bursts and thematic shifts, while the Bibliometrix package in R (v4.2.1) was utilized for descriptive statistics, Bradford’s Law analysis, and network visualizations. To avoid distortion of the maps, thresholds were set at a minimum of 20 publications and 50 citations for inclusion. To improve network readability and avoid excessive visual density, articles involving more than 25 authors, institutions, or countries were excluded from collaboration network visualizations. This threshold was applied for visualization clarity only and not for quantitative analyses.

Descriptive statistics were calculated to characterize publication growth, authorship patterns, and citation trends. Annual publication counts were summarized to illustrate temporal trends. Simple linear regression was applied as a descriptive tool to indicate the overall direction of publication growth and was not intended to model non-linear innovation dynamics or provide predictive inference. Data for 2025 represent a partial publication year. Author productivity and citation counts were calculated based on publications included in the BPH-specific dataset. In contrast, h-index values were obtained from Web of Science (WoS) author profiles and therefore represent overall academic impact rather than BPH-specific research performance. Country-level productivity was assessed using absolute publication counts to describe overall research output. Bradford’s Law was applied to identify core journals in the field, and significance was defined at *p* < 0.05.

## Results

### General characteristics of the dataset

Between January 2016 and August 2025, a total of 2,613 publications on surgical management of BPH were identified. Among these, 2,161 were original articles and 452 were reviews, published across 394 different journals. The annual growth rate was calculated at 2.9%, confirming a steady increase in research output during the study period. Each paper had an average of 3.97 authors, with a mean citation rate of 10.7 per document. The dataset contained 24,415 references, while 24.0% of publications involved international collaborations (Table 1).

**Table 1:** Main information about analysis

Description	Results
Main information about data	
Timespan	January 2016-August 2025
Sources (Journals, Books, etc.)	394
Documents	2613
Annual Growth Rate %	2.88
Document Average Rate	3.97
Average Citations per doc	10.66
References	24,415
Document Contents	
Keywords Plus (ID)	1822
Author’s Keywords	2994
Authors	
Authors	10,278
Authors of Single authored-docs	26
Authors Collaboration	
Single-authored docs	28
Co-Author per Doc	7.08
International co-authorship %	24.01
Document Types	
Article	2161
Review	452

### Annual publication trends

**Fig. 2:**
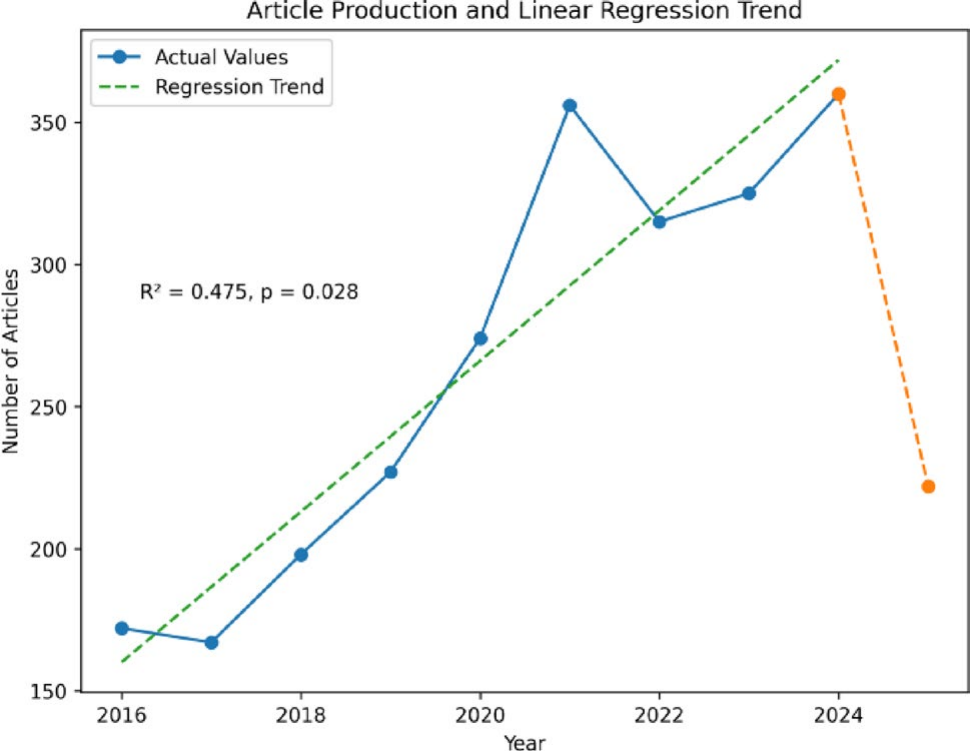
Article production and linear regression trend in the surgical management of benign prostatic hyperplasia (2016–2025). Annual publication output is shown with solid lines for complete publication years (2016–2024). The dashed segment represents 2025, which reflects partial-year data at the time of analysis. The dashed regression line summarizes the overall direction of publication growth and is included for descriptive purposes only

The temporal distribution of publications demonstrated a gradual but overall increase over the past decade. As illustrated in Fig. 2, the annual number of articles rose from 172 in 2016 to a peak of 360 in 2024, with a modest decline observed in 2025 (222 publications), which likely reflects partial-year data capture rather than a true reduction in research activity. A simple linear regression was applied for descriptive purposes only to summarize the overall direction of publication growth, yielding an average annual increase of approximately 16.9 articles (*p* = 0.028; R² = 0.475).

### Author collaboration networks

**Fig. 3:**
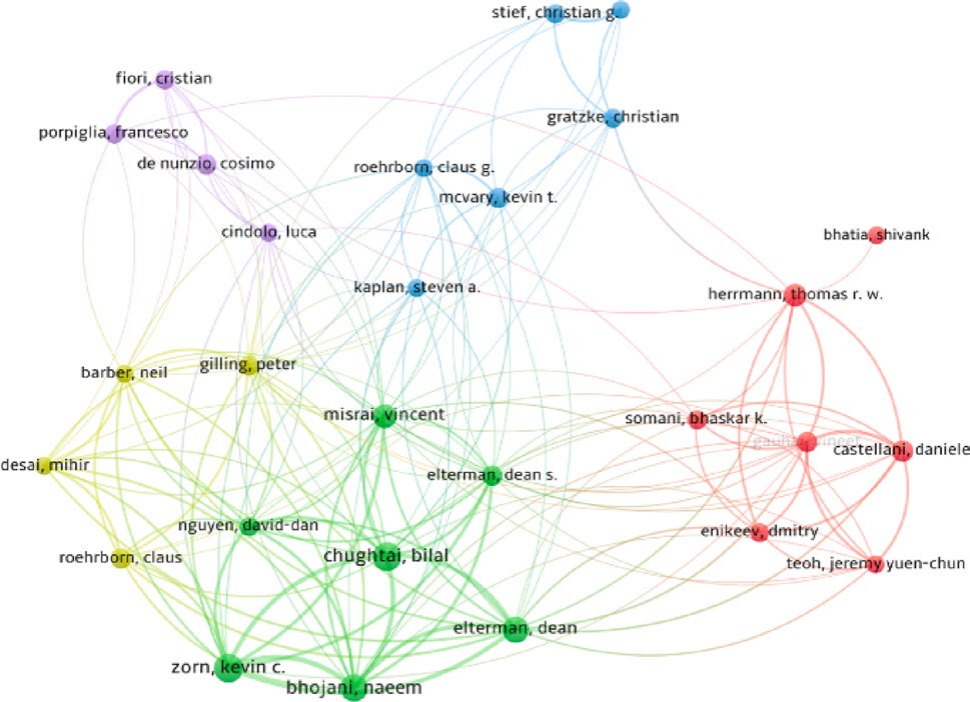
Author Co-authorship Visualization Map **(**Articles with more than 25 authors were excluded, and the full counting method was selected. A minimum threshold of 20 publications and 50 citations was set for each author. These criteria were met by 30 authors. Weighting was based on the number of publications, and the size of the circles on the map represents the number of articles.)

The dataset comprised contributions from 10,278 authors, reflecting a highly collaborative research community. Figure 3 shows the co-authorship visualization map, which demonstrates several dense clusters representing established international collaborations. The most prolific authors were Kevin C. Zorn (78 publications, 1,170 citations), Bilal Chughtai (74 publications, 805 citations), Naeem Bhojani (69 publications, 881 citations), Dean Elterman (63 publications, 890 citations), and Thomas RW Herrmann (47 publications, 763 citations). Citation analysis confirmed that many of these investigators also ranked among the most frequently cited, with multiple authors attaining h-indices above 25 (Table 2). In addition to their high publication counts, these authors consistently ranked among the most cited, with Zorn and Elterman demonstrating the strongest combined productivity and impact.

**Table 2:** Top ten authors

Author	Documents			Citations	Google Scholar h-index
	T	OP	R		
Kevin C. Zorn	78	69	9	1170	44*
Bilal Chughtai	74	58	16	805	43
Naeem Bhojani	69	61	8	881	35
Dean Elterman	63	55	8	890	34
Thomas Herrmann	47	36	11	763	60
Vincent Misrai	45	23	22	627	35
Bhaskar Kumar Somani	39	26	13	433	75
Daniele Castellani	37	28	9	357	30
Cosimo De Nunzio	32	24	8	396	58
Jeremy Yuen-Chun Teoh	32	18	14	303	45

*T* Total, *OP* Original Paper, *R* Review

*As it could not be reaches from Google Scholar, this h-index is based on Web of Scince

### Country-level contributions and collaboration

**Fig. 4:**
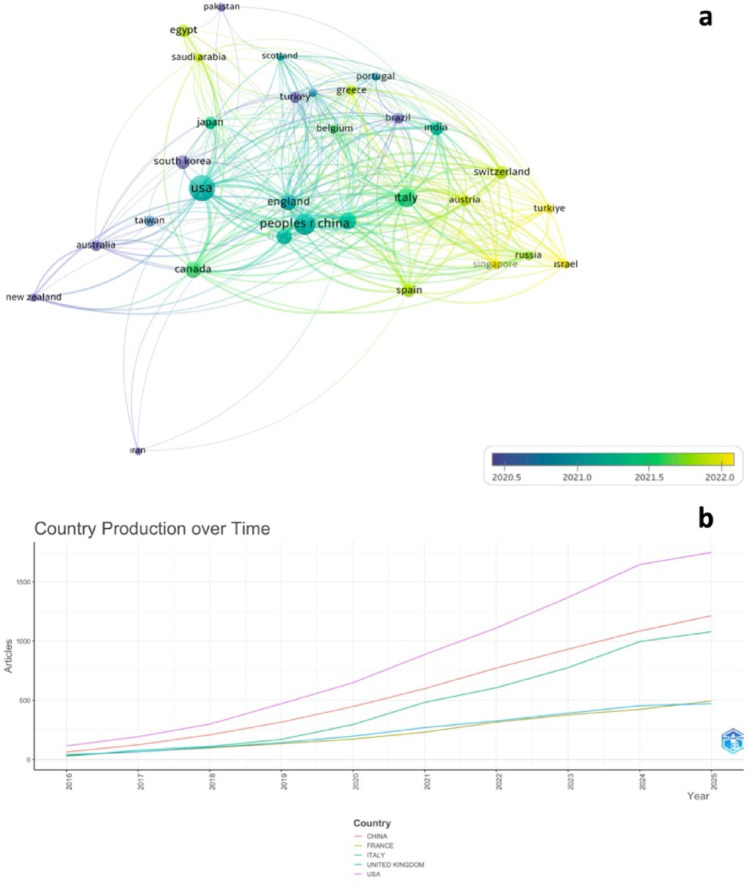
**a** Country Co-authorship visualization map (Articles with more than 25 different countries were excluded, and the full counting method was selected. A minimum threshold of 20 publications and 50 citations was set for each country. These criteria were met by 31 countries. Weighting was based on the number of publications, and the size of the circles on the map represents the number of articles.) **b** Country production over time

At the national level, the United States dominated both in productivity and influence, contributing 1,747 publications and accumulating 7,691 citations. China ranked second with 1,213 publications and 3,277 citations, followed by Italy (1,078 publications, 2,890 citations), France (494 publications, 958 citations), and the United Kingdom (471 publications, 1,452 citations) (Table 3). The co-authorship visualization map (Fig. 4a) revealed strong intercontinental collaborations, particularly between North America and Europe, while Fig. 4b illustrates the rapid expansion of Asian contributions, most notably from China and South Korea, over the last decade. Although the USA produced the highest number of citations (7,691), several European countries demonstrated higher citation averages relative to their overall output.

**Table 3:** Top ten countries

Country	Documents			Citations
	T	OP	*R*	
USA	1747	1470	277	7691
China	1213	995	218	3277
Italy	1078	824	254	2890
France	494	388	106	958
United Kingdom	471	302	169	1452
Germany	432	368	64	1672
Canada	407	348	59	1249
South Korea	332	278	54	983
Japan	261	238	23	763
Turkey	232	218	14	402

*T* Total, *OP* Original Paper, *R* Review

### Institutional networks

**Fig. 5:**
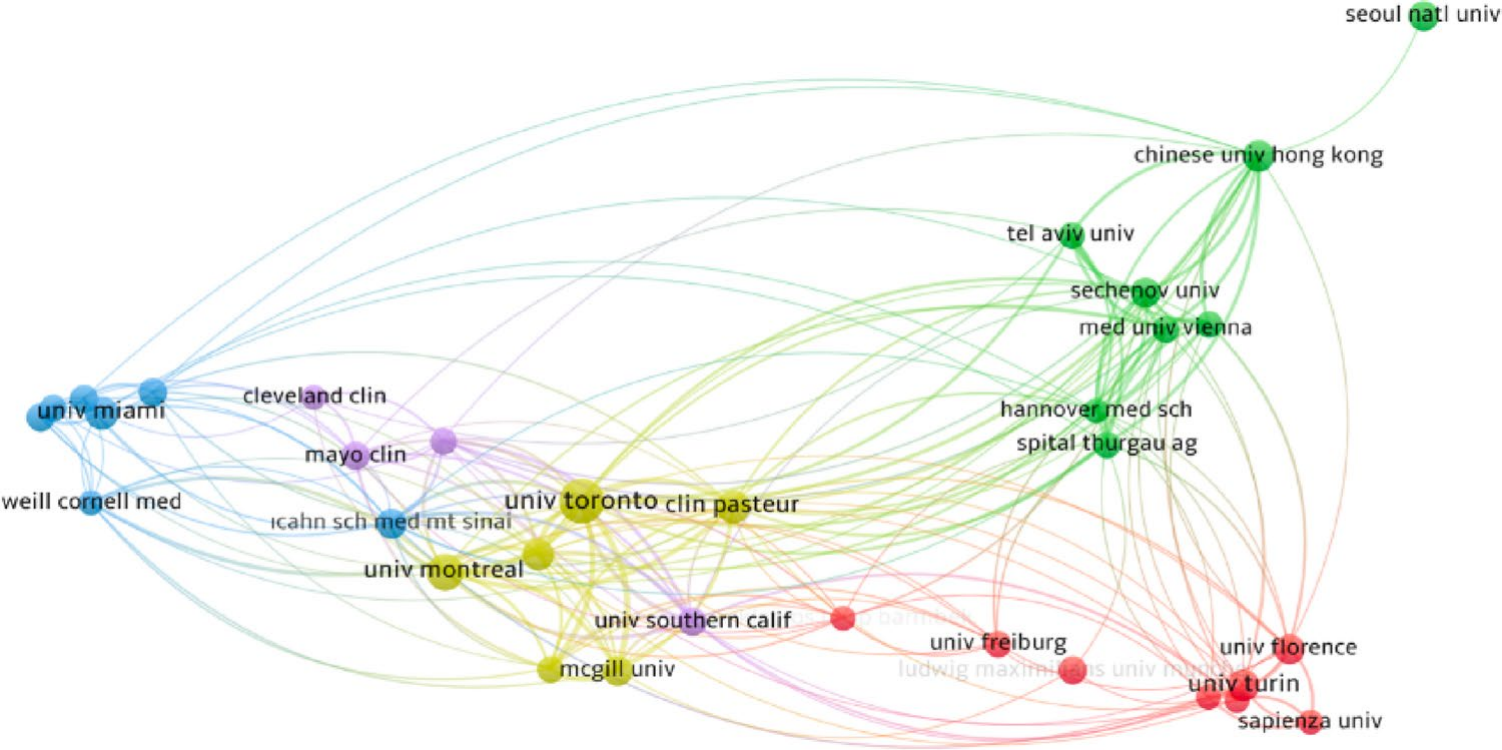
Institution Co-authorship Visualization Map (Articles with authors from more than 25 different organizations were excluded, and the full counting method was selected. A minimum threshold of 20 publications and 50 citations was set for each institution. These criteria were met by 36 institutions. Weighting was based on the number of publications, and the size of the circles on the map represents the number of articles.)

Institutional mapping showed an important role of a limited number of high-volume centers in advancing the literature on surgical management of BPH. As shown in Fig. 5, a total of 36 institutions met the predefined thresholds (≥ 20 publications and ≥ 50 citations) and were included in the co-authorship visualization map. The University of Toronto ranked first with 111 publications and 1,207 citations, followed by the University of Miami (70 publications, 566 citations), University of Montreal (60 publications, 911 citations), Seoul National University (55 publications, 586 citations), and Clinique Pasteur, France (50 publications, 716 citations) (Table 4). Other institutions with strong dual performance included Weill Cornell Medical College (49 publications, 989 citations) and the University of Florence (50 publications, 674 citations). Canadian universities were particularly prominent, with Toronto, Montreal, and McGill all represented among the top ten institutions.

**Table 4:** Top ten organizations

Organization	Documents	Citations	Country
Univ Toronto	111	1207	Canada
Univ Miami	70	566	USA
Univ Montreal	60	911	Canada
Seoul Natl Univ	55	586	South Korea
Clin Pasteur	50	716	France
Univ Florence	50	674	Italy
Sechenov Univ	49	586	Russia
Weill Cornell Med	49	989	USA
CollChinese Univ	47	354	China
Hong Kong Mcgill Univ	45	492	Canada

### Journals and source analysis

**Fig. 6:**
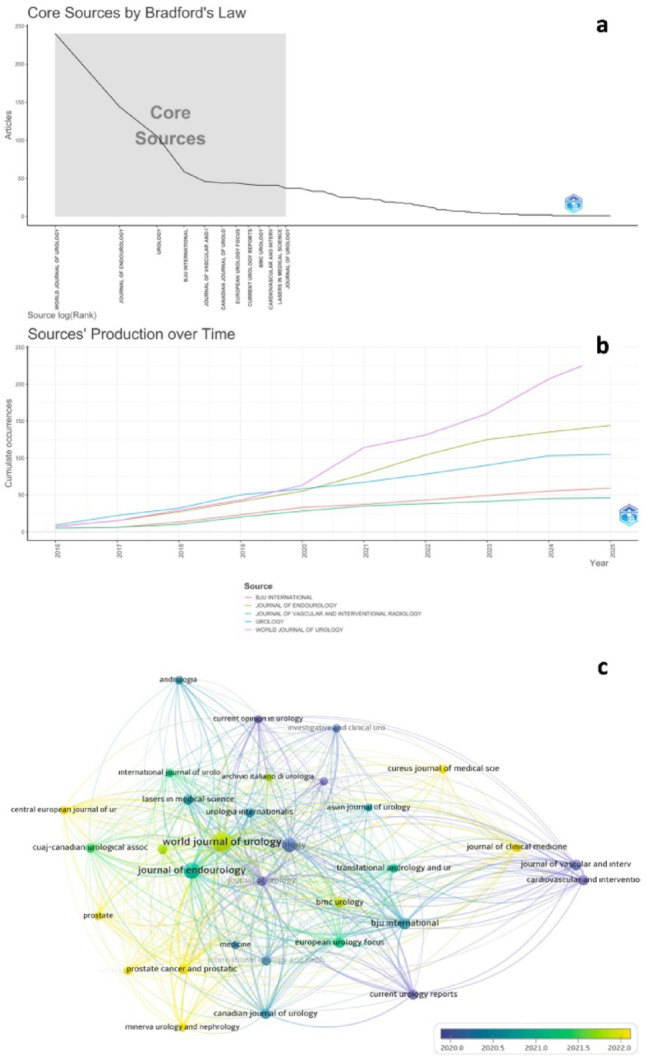
**a** Bradford’s Law. **b** Source’s production over time. **c** Citation Sources Visualization Map (A minimum threshold of 20 publications and 50 citations was set for each journal. These criteria were met by 32 journals. Weighting was based on the number of publications, and the size of the circles on the map represents the number of articles.)

Publication sources were widely distributed, but Bradford’s Law confirmed a concentration of output within a small core of journals. As shown in Fig. 6a–c, The World Journal of Urology published the largest number of articles (240) and accumulated 2,589 citations. The Journal of Endourology followed with 144 publications and 1,774 citations, while Urology (105 publications, 1,774 citations) and BJU International (59 publications, 1,664 citations) also contributed substantially (Table 5). As predicted by Bradford’s Law, these core journals concentrated both high productivity and high citation rates, serving as the primary platforms for dissemination of surgical BPH management. Citation analysis reinforced the influence of these journals, which together accounted for the majority of highly cited works in the field (Table 5).

**Table 5:** Top ten sources

Source	Documents			Citations	WOS index	WOS quartile
	T	OP	*R*			
World Journal of Urology	240	206	34	2589	SCIE	Q2
Journal Of Endourology	144	133	11	1774	SCIE	Q2
Urology	105	97	8	1774	SCIE	Q2
BJU International	59	51	8	1664	SCIE	Q1
Journal of Vascular and Interventional Radiology	46	42	4	1026	SCIE	Q2
Canadian Journal of Urology	44	40	4	773	SCIE	Q2
European Urology Focus	44	22	22	700	SCIE	Q1
Current Urology Reports	42	28	14	540	SCIE	Q2
BMC Urology	41	40	1	282	SCIE	Q3
Cardiovascular and Interventional Radiology	41	34	7	980	SCIE	Q2

*T* Total, *OP* Original Paper, *R* Review, *SCIE* Scientific Citation Index Expanded, *WOS* Web of Science

### Highly cited works

The ten most frequently cited papers are listed in Table 6. The most influential article was Roehrborn (2017), published in the Canadian Journal of Urology, which has been cited 258 times. Other landmark works included Foster (2018, Journal of Urology) with 234 citations and Lerner (2021, Journal of Urology) with 221 citations. These studies encompassed diverse themes, ranging from the validation of minimally invasive therapies to randomized controlled trials of HoLEP and TURP, as well as investigations introducing prostatic artery embolization.

**Table 6:** Top ten documents

Document	Citations	Doi	Journal
Roehrborn (2017) [23]	258	N/A	Canadian Journal of Urology
Foster (2018) [35]	234	10.1016/j.juro.2018.05.048	Journal of Urology
Lerner (2021) [34]	221	10.1097/JU.0000000000002184	Journal of Urology
Abt (2018) [28]	206	10.1136/bmj.k2338	BMJ
Carnevale (2016) [29]	203	10.1007/s00270-015–1202-4	Cardiovascular and Interventional Radiology
Thomas (2016) [31]	200	10.1016/j.eururo.2015.07.054	European Urology
Mcvary (2016) [22]	200	10.1016/j.juro.2015.10.181	Journal of Urology
Ray (2018) [33]	199	10.1111/bju.14,249	BJU International
Gilling (2018) [30]	180	10.1016/j.juro.2017.12.065	Journal of Urology
Mcvary (2019) [32]	176	10.1016/j.urology.2018.12.041	Urology

### Keyword analysis

**Fig. 7:**
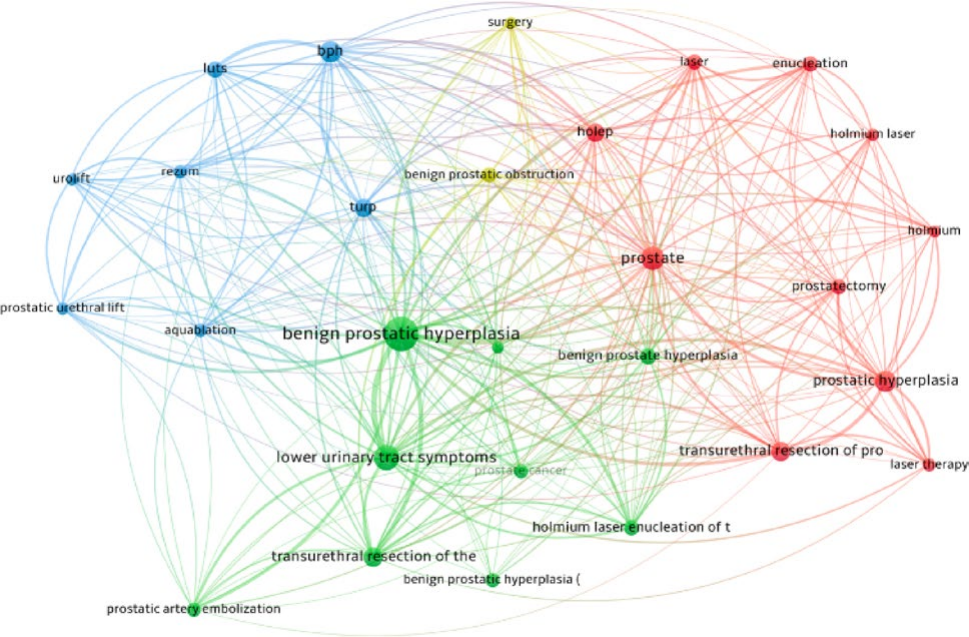
Co-ocurrence analysis of author keywords (The visualization is based on the total event count, and only author keywords that appeared at least 50 times have been included. This criterion was met by 28 author keywords. The visualization map is provided.)

A total of 2,994 author keywords were identified, with 28 meeting the threshold of appearing at least 50 times. The most common terms included “benign prostatic hyperplasia” (1,019 occurrences), “transurethral resection” (744), “holmium laser enucleation” (452), and “complications” (448) (Table 7). Figure 7 illustrates the keyword co-occurrence map, which revealed three major thematic clusters: (1) traditional resection-based procedures and their complications, (2) laser and enucleation-based technologies such as HoLEP and ThuLEP, and (3) emerging minimally invasive interventions including Aquablation, Rezūm, and prostatic artery embolization.

**Table 7:** Top ten keywords

Keyword	Occurrences
benign prostatic hyperplasia	1019
transurethral resection	744
hyperplasia	608
urinary-tract symptoms	597
holmium laser enucleation	452
complications	448
management	389
lower urinary tract symptoms	376
prostate	367
bph	309

### Thematic evolution

**Fig. 8:**
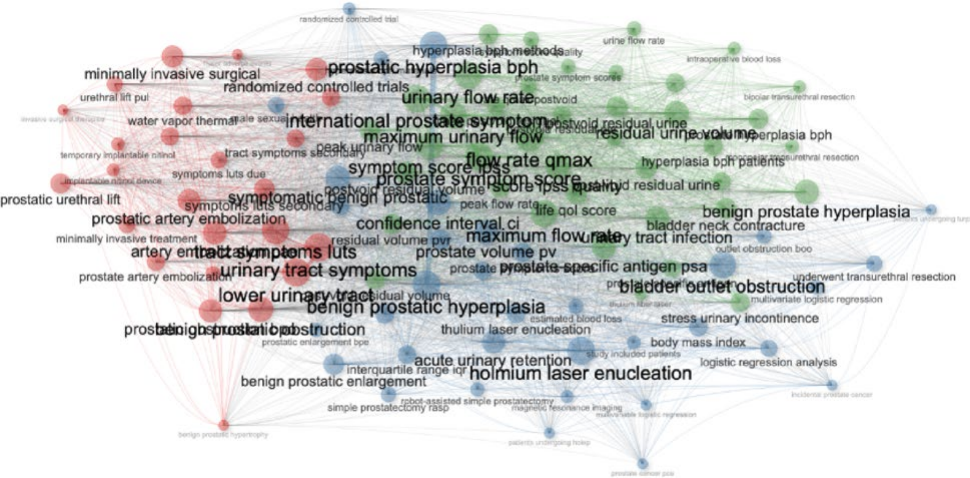
Thematic map (Field: abstract, N-Gram: Trigram, Word Stemming: No)

Thematic analysis provided further insights into how research priorities have shifted over time. As depicted in Fig. 8, early literature was dominated by TURP-related topics and classical resection techniques. From the mid-2010s onwards, however, attention increasingly shifted towards laser enucleation strategies, particularly HoLEP and ThuLEP, alongside novel minimally invasive procedures. Citation burst analysis identified recent surges in terms such as “robot-assisted simple prostatectomy,” “water vapor therapy,” and “transient urinary incontinence.” These bursts signal both the clinical uptake of innovative technologies and a growing focus on patient-centered functional outcomes, particularly continence and quality of life.

## Discussion

This bibliometric analysis reveals the dynamic evolution of research in the surgical management of BPH, reflecting both clinical necessity and technological innovation. With over 2,600 publications between 2016 and 2025 and an annual growth rate of 2.88%, the upward trend in research output illustrates sustained scholarly interest, especially in the context of rising global prevalence and treatment demands for BPH [1, 2, 13].

The increasing volume of publications (rising from 172 in 2016 to 360 in 2024) mirrors patterns seen across urological research domains, as surgical strategies grow more complex and individualized [14]. This expansion corresponds with the widespread adoption of laser-based and minimally invasive technologies, which have progressively challenged the dominance of traditional transurethral resection of the prostate (TURP). Our keyword and thematic analyses demonstrate a marked transition from resection-based procedures to enucleation techniques such as holmium laser enucleation (HoLEP) and thulium laser enucleation (ThuLEP), as well as to newer options like Aquablation, Rezūm, and prostatic artery embolization (PAE). These findings echo recent bibliometric studies that identify endoscopic enucleation of the prostate (EEP) as a major research hotspot [10]. While our analysis primarily captured mainstream surgical management strategies, isolated reports on extreme conditions such as giant prostatic hyperplasia underscore the breadth of clinical scenarios represented in the literature [15]. The rising dominance of anatomic endoscopic enucleation is consistent with recent consensus efforts to standardize this technique, most prominently the Delphi study conducted by the ESUT group [16]. This evolution aligns with global systematic reviews highlighting the decline of resection-based techniques and the emergence of anatomic enucleation as a dominant theme in BPH surgery [17, 18]. With their demonstrated efficacy and favorable safety profiles, these approaches have gained endorsement in contemporary clinical guidelines [19, 20].

The shift from TURP to enucleation strategies is not only bibliometrically evident but also clinically justified, as anatomic enucleation has demonstrated superior safety and durability in expert reviews [21]. Their growing research presence likely reflects increasing clinical confidence, enhanced surgical training, and the dissemination of comparative data positioning them as viable alternatives to open simple prostatectomy. Meanwhile, the ascent of office-based modalities such as Rezūm and UroLift illustrates the profession’s pivot toward techniques that prioritize patient comfort, reduced anesthesia requirements [7, 22, 23].

High-impact authors (including Zorn, Chughtai, Elterman, and Bhojani) played central roles in advancing these innovations. Their citation metrics, coupled with institutional leadership from centers like the University of Toronto and University of Montreal, underscore the importance of concentrated academic ecosystems. These findings are consistent with bibliometric studies in urology showing that top-tier academic centers often serve as incubators for surgical innovation [24, 25]. The density of international collaborations (reflected in our co-authorship maps) also speaks to the multidisciplinary and global nature of BPH surgery research, which increasingly requires coordination between urologists, radiologists, engineers, and data scientists.

From a geographic standpoint, the United States remains the dominant contributor, responsible for nearly 67% of all citations, followed by China and Italy. This distribution reflects national disparities in research funding, healthcare infrastructure, and academic-industrial partnerships. However, the citation efficiency of countries like France and the United Kingdom (despite smaller publication volumes) suggests a sustained focus on methodological quality and impact. This phenomenon has been previously noted in global urology literature and may reflect differing academic priorities and publication strategies [26, 27].

Our journal analysis confirmed a classic Bradford’s Law distribution, with a small group of journals (including the World Journal of Urology, Journal of Endourology, Urology, and BJU International) hosting a majority of influential articles. These core journals not only offer visibility but also shape the research agenda by curating studies on emerging therapies and practice-changing trials. For example, landmark studies on PAE [28, 29], Aquablation [30], and GreenLight laser [31], and Rezūm [22, 32] have significantly influenced contemporary thinking and guideline development.

Our analysis of keyword co-occurrence revealed three dominant clusters: classical resection techniques, laser-based enucleation, and emerging minimally invasive interventions. This thematic structure is reinforced by the results of citation burst detection, which highlighted recent surges in terms like “robot-assisted simple prostatectomy,” “water vapor therapy,” and “transient urinary incontinence.” These topics not only reflect technical innovation but also a shifting focus toward functional outcomes and patient-centered care, a growing concern in the aging male population undergoing BPH surgery [33, 34].

Despite its strengths, this study is not without limitations. Restricting the search to the Web of Science Core Collection may have excluded relevant articles indexed in other databases like Scopus or Embase. Additionally, the exclusion of non-English language publications may have inadvertently limited representation from certain regions, despite growing contributions from Asia and Eastern Europe. Our analysis also focused on quantitative bibliometric indicators, which, while robust, cannot fully assess the methodological quality or clinical impact of individual studies. Publication trends in innovation-driven surgical fields are inherently non-linear and may be influenced by technological adoption cycles, guideline updates, and external events such as the COVID-19 pandemic. Therefore, the linear trend presented in this study should be interpreted as a descriptive summary of long-term publication growth rather than a detailed model of innovation dynamics. Authorship position-based analyses (e.g., first or senior authorship) were not performed due to limitations in accurately identifying leadership roles within large bibliometric datasets, including author name disambiguation and heterogeneous authorship conventions. Collaboration and institutional network analyses required threshold-based exclusions to ensure visual interpretability. As a result, large multicenter or consortium-based studies with extensive author lists may have been underrepresented in network visualizations, potentially biasing the apparent collaboration structure. However, these exclusions did not affect overall publication volume, citation metrics, or thematic analyses. Author-level metrics such as the h-index reflect cumulative academic impact across all research domains and may not accurately represent field-specific influence within BPH research. Similarly, institutional productivity metrics may be disproportionately driven by a limited number of highly prolific researchers rather than broad, team-based research activity. These factors should be considered when interpreting author and institutional rankings. Country-level productivity was evaluated using absolute publication counts without normalization for population size or the number of practicing urologists. As a result, comparisons across countries should be interpreted cautiously, as larger countries may appear disproportionately dominant. Normalized indicators could provide additional context but were beyond the scope of the present analysis. Furthermore, the inability to distinguish between author roles in collaborative papers may slightly distort perceived individual contributions.

### Future research directions and emerging technologies

As newer technologies, including robotic platforms and artificial intelligence-based tools, begin to appear in clinical research, bibliometric analyses may help describe how technological approaches are reflected in the evolving literature. Such observations can provide context for emerging areas of interest and may inform considerations related to training needs and resource planning. These developments appear consistent with a gradual movement toward more individualized and minimally invasive strategies in urology, with surgical BPH management representing one area where these changes are becoming visible.

## Conclusion

This bibliometric study describes how research on the surgical management of BPH has evolved worldwide over the past decade. Overall publication activity has increased steadily, accompanied by a growing diversity of surgical techniques and research themes. By examining publication patterns, collaboration networks, and thematic changes, the study provides an overview of current research priorities and highlights areas where further investigation may be warranted. These findings may help clinicians and researchers better understand the structure and direction of the contemporary surgical BPH literature.

## Supplementary Information

Below is the link to the electronic supplementary material.

Supplementary Material 1

## Data Availability

The data that support the findings of this study are available from the corresponding author upon reasonable request.

## References

[1] Wei H, Zhu C, Huang Q et al (2025) Global, regional, and national burden of benign prostatic hyperplasia from 1990 to 2021 and projection to 2035. BMC Urol 25:34. 10.1186/s12894-025-01715-9 39972318 10.1186/s12894-025-01715-9 PMC11837592

[2] Lee SWH, Chan EMC, Lai YK (2017) The global burden of lower urinary tract symptoms suggestive of benign prostatic hyperplasia: a systematic review and meta-analysis. Sci Rep 7(1):7984. 10.1038/s41598-017-06628-8 28801563 10.1038/s41598-017-06628-8 PMC5554261

[3] Chen X, Yang S, He Z et al (2025) Comprehensive analysis of the global, regional, and national burden of benign prostatic hyperplasia from 1990 to 2021. Sci Rep 15(1):5644. 10.1038/s41598-025-90229-3 39955408 10.1038/s41598-025-90229-3 PMC11830103

[4] Goel A (2019) Lower urinary tract symptoms and benign prostatic hyperplasia: from research to bedside. Indian J Med Res 149(5):684–685. 10.4103/ijmr.IJMR_323_19

[5] Guldibi F, Altunhan A, Aydın A et al (2023) What is the effect of laser anatomical endoscopic enucleation of the prostate on the ejaculatory functions? A systematic review. World J Urol 41(12):3493 − 501. 10.1007/s00345-023-04660-0 37921935 10.1007/s00345-023-04660-0

[6] Wang R, Gong X, Shu T, Yang S (2024) Laser application in surgical treatment of benign prostatic hyperplasia. J Contemp Med Pract 6(10):57–60. 10.53469/jcmp.2024.06(10).11

[7] Nguyen D-D, Li T, Ferreira R et al (2023) Ablative minimally invasive surgical therapies for benign prostatic hyperplasia: a review of Aquablation, Rezum, and transperineal laser prostate ablation. Prostate Cancer Prostatic Dis 27(1):22–28. 10.1038/s41391-023-00669-z 37081044 10.1038/s41391-023-00669-z

[8] Akgul B, Aydin A, Cakir OO et al (2025) Impact of minimally invasive surgical therapies on sexual function in benign prostatic hyperplasia: a systematic review. Minerva Urol Nephrol 77(4):459–71. 10.23736/s2724-6051.25.06374-8 40891476 10.23736/S2724-6051.25.06374-8

[9] Franco JVA, Tesolin P, Jung JH (2023) Update on the management of benign prostatic hyperplasia and the role of minimally invasive procedures. Prostate Int 11(1):1–7. 10.1016/j.prnil.2023.01.002 36910900 10.1016/j.prnil.2023.01.002 PMC9995694

[10] Lan X-D, Yu Z-Y, Jiang R et al (2025) Application trends and research hotspots of endoscopic enucleation of the prostate: a bibliometric and visualization analysis. World J Urol 43(1):140. 10.1007/s00345-024-05379-2 40009250 10.1007/s00345-024-05379-2

[11] Pritchard A (1969) Statistical bibliography or bibliometrics. J Doc 25:348–349

[12] Kuzior A, Sira M (2022) A bibliometric analysis of blockchain technology research using VOSviewer. Sustainability 14(13):8206. 10.3390/su14138206

[13] Chu F, Chen L, Guan Q et al (2025) Global burden of prostate cancer: age-period-cohort analysis from 1990 to 2021 and projections until 2040. World J Surg Oncol 23(1):98. 10.1186/s12957-025-03733-1 40114188 10.1186/s12957-025-03733-1 PMC11924780

[14] Majzoub A, Al Rumaihi K, Al Ansari A (2016) The world’s contribution to the field of urology in 2015: a bibliometric study. Arab J Urol 14(4):241–247. 10.1016/j.aju.2016.09.004 27900212 10.1016/j.aju.2016.09.004 PMC5122814

[15] Yilmaz K, Istanbulluoglu O, Guven S, Kilinc M (2006) Giant prostatic hyperplasia: case report. Int Urol Nephrol 38(3–4):587–589. 10.1007/s11255-006-9012-x 17043922 10.1007/s11255-006-9012-x

[16] Tunc L, Herrmann T, Guven S et al (2023) A Delphi consensus to standardize the technique of anatomical endoscopic enucleation of prostate: a study by ESUT endoscopic enucleation of prostate study group. World J Urol 41(9):2303–2309. 10.1007/s00345-023-04496-8 37421419 10.1007/s00345-023-04496-8

[17] Turney BW, Cornu JN, Schatteman P et al (2025) Evolution of the endoscopic surgical approach for benign prostatic obstruction in European countries. Eur Urol Focus (5):00070. 10.1016/j.euf.2025.03.014 10.1016/j.euf.2025.03.014 40189998

[18] Nettleton J, Jones P, Pietropaolo A et al (2019) The industrial revolution for the management of benign prostate obstruction: worldwide publication trends for surgical and medical therapies over the past two decades. Cent Eur J Urol 72(2):149–155. 10.5173/ceju.2019.1876 10.5173/ceju.2019.1876 PMC6715083 31482021

[19] Sandhu JS, Bixler BR, Dahm P et al (2024) Management of lower urinary tract symptoms attributed to benign prostatic hyperplasia (BPH): AUA guideline amendment 2023. J Urol 211(1):11–9. 10.1097/ju.0000000000003698 37706750 10.1097/JU.0000000000003698

[20] Gravas S, Cornu JN, Gacci M, Gratzke C, Herrmann TRW, Mamoulakis C, Rieken M, Speakman MJ, Tikkinen, K A O (2025) EAU Guidelines on Management of Non-Neurogenic Male Lower Urinary Tract Symptoms (LUTS). European Association of Urology. https://uroweb.org/guidelines/management-of-non-neurogenic-male-luts. Accessed 30 Aug 2025

[21] Gómez-Sancha F (2021) The constant search for the greater good: evolving from TURP to anatomic enucleation of the prostate is a safe bet. World J Urol 39(7):2401–2406. 10.1007/s00345-021-03637-1 33625568 10.1007/s00345-021-03637-1

[22] McVary KT, Gange SN, Gittelman MC et al (2016) Minimally invasive prostate convective water vapor energy ablation: a multicenter, randomized, controlled study for the treatment of lower urinary tract symptoms secondary to benign prostatic hyperplasia. J Urol 195(5):1529–1538. 10.1016/j.juro.2015.10.181 26614889 10.1016/j.juro.2015.10.181

[23] Roehrborn CG, Barkin J, Gange SN et al (2017) Five year results of the prospective randomized controlled prostatic urethral L.I.F.T. Study. Can J Urol 24(3):8802–8813 28646935

[24] Paniagua Cruz A, Zhu KY, Ellimoottil C, Dauw CA, Sarma A, Skolarus TA (2020) Characterizing the benign prostatic hyperplasia literature: a bibliometric analysis. Urology 136:202–211. 10.1016/j.urology.2019.11.033 31801683 10.1016/j.urology.2019.11.033

[25] An JY, Baiocco JA, Rais-Bahrami S (2018) Trends in the authorship of peer reviewed publications in the urology literature. Urol Pract 5(3):233–239. 10.1016/j.urpr.2017.03.008 29744377 10.1016/j.urpr.2017.03.008 PMC5937928

[26] Willis DL, Bahler CD, Neuberger MM, Dahm P (2011) Predictors of citations in the urological literature. BJU Int 107(12):1876–1880. 10.1111/j.1464-410x.2010.10028.x 21332629 10.1111/j.1464-410X.2010.10028.x

[27] Kulkarni AV, Busse JW, Shams I (2007) Characteristics associated with citation rate of the medical literature. PLoS One 2(5):e403. 10.1371/journal.pone.0000403 17476325 10.1371/journal.pone.0000403 PMC1852582

[28] Abt D, Hechelhammer L, Müllhaupt G et al (2018) Comparison of prostatic artery embolisation (PAE) versus transurethral resection of the prostate (TURP) for benign prostatic hyperplasia: randomised, open label, non-inferiority trial. BMJ 361:k2338. 10.1136/bmj.k2338 29921613 10.1136/bmj.k2338 PMC6006990

[29] Carnevale FC, Iscaife A, Yoshinaga EM, Moreira AM, Antunes AA, Srougi M (2016) Transurethral resection of the prostate (TURP) versus original and perfected prostate artery embolization (PAE) due to benign prostatic hyperplasia (BPH): preliminary results of a single center, prospective, urodynamic-controlled analysis. Cardiovasc Intervent Radiol 39(1):44–52. 10.1007/s00270-015-1202-4 26506952 10.1007/s00270-015-1202-4

[30] Gilling P, Barber N, Bidair M et al (2018) WATER: a double-blind, randomized, controlled trial of Aquablation(®) vs transurethral resection of the prostate in benign prostatic hyperplasia. J Urol 199(5):1252–1261. 10.1016/j.juro.2017.12.065 29360529 10.1016/j.juro.2017.12.065

[31] Thomas JA, Tubaro A, Barber N et al (2016) A multicenter randomized noninferiority trial comparing GreenLight-XPS laser vaporization of the prostate and transurethral resection of the prostate for the treatment of benign prostatic obstruction: two-yr outcomes of the GOLIATH study. Eur Urol 69(1):94–102. 10.1016/j.eururo.2015.07.054 26283011 10.1016/j.eururo.2015.07.054

[32] McVary KT, Rogers T, Roehrborn CG (2019) Rezūm water vapor thermal therapy for lower urinary tract symptoms associated with benign prostatic hyperplasia: 4-year results from randomized controlled study. Urology 126:171–179. 10.1016/j.urology.2018.12.041 30677455 10.1016/j.urology.2018.12.041

[33] Ray AF, Powell J, Speakman MJ et al (2018) Efficacy and safety of prostate artery embolization for benign prostatic hyperplasia: an observational study and propensity-matched comparison with transurethral resection of the prostate (the UK-ROPE study). BJU Int 122(2):270–282. 10.1111/bju.14249 29645352 10.1111/bju.14249

[34] Lerner LB, McVary KT, Barry MJ et al (2021) Management of lower urinary tract symptoms attributed to benign prostatic hyperplasia: AUA GUIDELINE PART II-surgical evaluation and treatment. J Urol 206(4):818–826. 10.1097/ju.0000000000002184 34384236 10.1097/JU.0000000000002184

[35] Foster HE, Barry MJ, Dahm P et al (2018) Surgical management of lower urinary tract symptoms attributed to benign prostatic hyperplasia: AUA guideline. J Urol 200(3):612–619. 10.1016/j.juro.2018.05.048 29775639 10.1016/j.juro.2018.05.048

