# Supplementary material for: Characterizing the academic literature on surgical management of benign prostatic hyperplasia: A bibliometric analysis
Source: World J Urol. 2026 Feb 11;44(1):159. doi: 10.1007/s00345-026-06257-9 (PMC12894143; doi:10.1007/s00345-026-06257-9)
Supplement: Supplementary file 1 — Supplementary Material 1 [file 345_2026_6257_MOESM1_ESM.docx]

**Supplement 1. Search strategy for Web of Science Core Collection (18/08/2025)**

| Link: https://www.webofscience.com/wos/woscc/summary/a2dc89b4-9ed6-4f17-b8a3-fb0267aaedbc-0174c30989/relevance/1 |
| --- |
| Search query: (TS=(“benign prostat* hyperpl*” OR “benign prostat* hypert*” OR “benign prostat* obstr*” OR “prostat* hyperplas*” OR “prostat* obstr*” OR “prostat* enlarge*” OR “benign prost*” OR “lower urin* tract sympt*”)) AND (TS=( “anatom* endoscopic enucleation of the prostate” OR “aquablation” OR “B-TUEP” OR “B-TURP” OR “B-TUVP” OR “bipolar enucleation” OR “bipolar enucleation of the prostate” OR “bipolar transurethral enucleation of the prostate” OR “bipolar transurethral vaporisation of the prostate” OR “bipolar transurethral vaporization of the prostate” OR “BipolEP” OR “Butterfly prostat* retraction” OR “DiLEP” OR “diode laser enucleation of the prostate” OR “endoscopic enucleation of the prostate” OR “GreenLEP” OR “greenlight laser” OR “HoLAP” OR “HoLEP” OR “holmium laser ablation of the prostate” OR “holmium laser enucleation of the prostate” OR “holmium laser resection of prostate” OR “HoLRP” OR “iTind” OR “LAEEP” OR “laparoscopic adenomectomy” OR “laparoscopic simple prostatectomy” OR “laser anatomical endoscopic enucleation of the prostate” OR “laser enucleation” OR “laser vaporization” OR “laser vaporization” OR “minimal invasive simple prostatectomy” OR “minimal invasive prostatectomy” OR “minimal invasive surgical therapy” OR “minimal invasive therapy” OR “minimally invasive surgery” OR “open simple prostatectomy” OR “Optilume BPH Catheter system” OR “photoselective vaporation of the prostate” OR “prostatic artery embolization” OR “prostatic urethral lift” OR “PU lift” OR “Rezum” OR “Rezūm” OR “robot-assisted simple prostatectomy” OR “robotic simple prostatectomy” OR “Robotic waterjet treatment” OR “Temporary implant* nitinol device” OR “ThuFLEP” OR “ThuLEP” OR “Thulium enucleation” OR “thulium fiber laser enucleation of the prostate” OR “Thulium laser enucleation of the prostate” OR “Thulium laser vaporesection of the prostate” OR “ThuVARP” OR “transurethral bipolar plasma resection of the prostate” OR “Transurethral incision of the prostate” OR “Transurethral microwave therapy” OR “Transurethral needle ablation” OR “Transurethral resection of the prostate” OR “Transurethtal vaporesection of the prostate” OR “TUIP” OR “TUMT” OR “TUNA” OR “TURP” OR “TUVRP” OR “Urethral lift” OR “Urolift” OR “Water vapor therapy” OR “Water vapor thermal therapy” OR “Water vapour therapy” OR “XFLO Expander system” OR “Zenflow spring system”)) |
